# Covert Channel Communication as an Emerging Security Threat in 2.5D/3D Integrated Systems

**DOI:** 10.3390/s23042081

**Published:** 2023-02-13

**Authors:** Ivan Miketic, Krithika Dhananjay, Emre Salman

**Affiliations:** Department of Electrical and Computer Engineering, Stony Brook University, Stony Brook, NY 11794, USA

**Keywords:** covert channel, interposer integration, 3D integration

## Abstract

In this paper, first, a broad overview of existing covert channel communication-based security attacks is provided. Such covert channels establish a communication link between two entities that are not authorized to share data. The secret data is encoded into different forms of signals, such as delay, temperature, or hard drive location. These signals and information are then decoded by the receiver to retrieve the secret data, thereby mitigating some of the existing security measures. The important steps of covert channel attacks are described, such as data encoding, communication protocol, data decoding, and models to estimate communication bandwidth and bit error rate. Countermeasures against covert channels and existing covert channel detection techniques are also summarized. In the second part of the paper, the implications of such attacks for emerging packaging technologies, such as 2.5D/3D integration are discussed. Several covert channel threat models for 2.5D/3D ICs are also proposed.

## 1. Introduction

Covert channel communication, where an adversary uses various methods to communicate sensitive data between a secure and insecure compute element, has gained attention as a potent attack. This communication can be between two personal computers, two cores within a multi-core processor, or between a computer speaker and microphone. Typically, strict protocols, based on the principle of “security by isolation”, are used in modern microprocessors [1,2,3,4]. Security by isolation involves having separate security domains for compute elements with different security requirements, where the amount of shared resources is minimized [5,6]. One example of security by isolation is an air-gap system, where a device is physically isolated and incapable of connecting to other unsecured computers and networks [7,8]. The only way to transfer data to an air-gapped system is through a physical device, such as a universal serial bus (USB) stick, with only a few trusted users having access [9]. Attackers have developed covert channel communication, demonstrating that this isolation is not sufficient to stop information leakage, regardless of the access control protocols implemented.

A covert channel is a communication channel between two entities (sender and receiver) that are not authorized to transfer information [10]. A side-channel, however, is the leakage of information, due to a side effect of the implementation and the way the computer hardware is used [11]. Side-channels involve observing the physical parameters (such as temperature, supply current, execution time, etc.) of a device during normal operation, rather than exploiting a flaw in the design/hardware such as covert channels [12,13,14,15,16,17]. Side-channels typically leak cryptographic information, while covert channels are more general because there is an intentional transmission of data [18,19,20,21]. Some covert channels can operate remotely without the need for physical access or modification.

**Figure 1:**
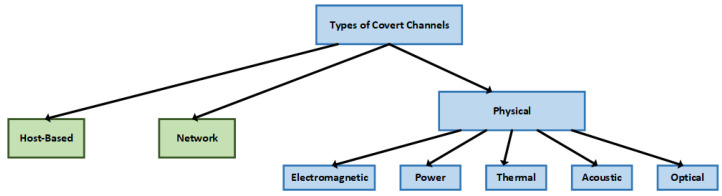
Classification of various covert channels.

**Figure 2:**
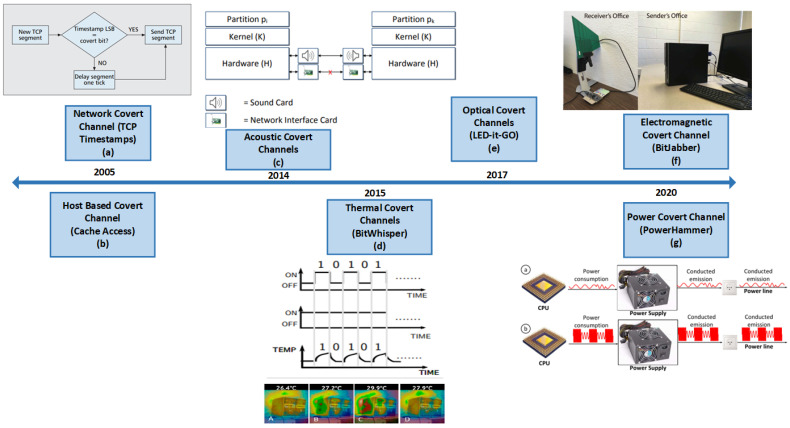
Timeline of various network-based, host-based and physical covert channels (**a**) TCP timestamps [28], (**b**) Cache Access [24], (**c**) acoustic channels [30], (**d**) Bitwhisper [33], (**e**) Led-it-GO [34], (**f**) BitJabber [31], (**g**) PowerHammer [32].

Covert channels can be broadly classified into three types: host-based, network-based and physical, as shown in Figure 1. Host-based covert channels typically involve manipulating the timing/storage properties of the host system [22]. An example of this type of covert channel involves one process probing the cache state by observing latency to determine if data was a hit or miss in the cache [23,24,25]. The hit/miss encodes the data being sent by the attacker. Another example of host-based channels includes covert channels through dynamic frequency scaling [26]. This work shows that manipulating the power governors, which scales the CPU frequency dynamically, can create a communication channel because CPU core frequency is generally available to user processes (through sysfs or/proc/cpuinfo) [26]. Network-based covert channels rely on manipulating some part of network traffic to establish communication between networked devices [10,27]. Various fields in the Open Systems Information (OSI) model are altered in order to transmit information; one example being modulating the least significant bit of the Transmission Control Protocol (TCP) timestamp field [28]. Finally, physical covert channels involve sending and encoding data through physical sources or side-channel signals (such as temperature, power, electromagnetic radiation, optical) [29]. Physical covert channels require some degree of proximity between transmitter and receiver elements in order to maintain a reliable communication channel. Since security enclaves, such as Intel Software Guard Extensions [3] and Arm TrustZone [1], are not sufficient for these types of covert channels, they typically pose a higher security threat and are the main focus of this work. A chronological timeline illustrating the developments of different covert channel techniques is shown in Figure 2. The development of network and cache-based covert channel attacks dates back to 2005 [24]. Physical covert channel attacks have been studied more recently, with an acoustic channel [30] introduced in 2014, and power/electromagnetic covert channels explored in 2020 [31,32].

Although there have been previous survey papers on covert channels, these works typically summarize covert channels as a whole (including network- and host-based channels), rather than focusing on physical covert channels [5,11,22]. Additionally, the discussion on physical covert channels is typically confined to air-gap systems instead of emerging threat models, such as covert channels between separate cores of a multi-core processor or even within the same core. The primary contributions of this paper are as follows:

- A summary of the general threat model and methodology involved in using a covert channel to leak secret information is provided.
- A detailed background on different types of physical covert channel attacks is provided.
- Modern countermeasures against physical covert channel attacks are discussed.
- A perspective on covert channels in emerging 2.5D/3D systems is provided.
- A novel attack model for a power covert channel that exploits the relatively accessible interposer layer in 2.5D systems is proposed.

The rest of this paper is organized as follows. Section 2 provides a background on the procedure of establishing a covert channel and describes the threat model assumed. Section 3 gives a survey of different types of physical covert channels and the existing state-of-the-art. Section 4 discusses various countermeasures to prevent and detect covert channels. Section 5 provides a perspective on upcoming challenges with covert channels in 2.5D/3D systems including covert channel detection. Finally Section 6 concludes the paper.

## 2. Background

The flow of establishing a covert channel, including the typically assumed threat model and different ways of encoding data, are covered in Section 2.1. Section 2.2 discusses how the metrics describing a covert channel, such as capacity and accuracy, are quantified.

### 2.1. Covert Channel Methodology

A covert channel attack first starts with identifying an exploit within the system that can leak information. For physical covert channels, this includes side-channel signals, such as thermal radiation, electromagnetic radiation, and power/supply current noise. After the potential exploit is identified, an appropriate modulation scheme is chosen to transmit the data, and an appropriate receiver must be established (temperature sensor, antenna, power probe, etc.). Typically, a preamble is sent before transmitting the secret data, in order to synchronize the transmission and signal the beginning of covert channel communication [35]. A preamble is a sequence of bits known by the receiver that helps determine the channel properties, such as carrier wave frequency and amplitude [36]. The following is an example of the full methodology of a thermal covert channel. Sensitive information that belongs to a core (referred to as a source) can be retrieved by an attacker who monitors the changes in temperature of another core within the same multi-core processor. Such an attack is possible provided that a temperature-based communication channel is established between the two cores, where energy intensive instructions are implemented on the transmitting core, thereby changing the internal temperature.

#### 2.1.1. Threat Model

A typical threat model for covert channel communication assumes two entities at any abstraction level (stand alone PCs or chiplets, or cores within a monolithic processor), where one device is the transmitting entity, while the other device is the receiving entity. It should be noted that the transmitting and receiving entities can be executing on the same host, two separate hosts that are connected via a network, or two separate hosts that are not connected to one another in any fashion [5]. These two systems are capable of communicating while thwarting the underlying system security policy (i.e., the communication between the two devices is unknown to the host). The access control policy can be described as the following: the transmitting device has access to sensitive data (for example, by operating in a secure zone), but the transfer of data to the receiving device is not allowed. The attack model assumes that malicious code is able to execute on the transmitting entity in order to encode and transmit the data through various side-channel signals (thermal, EM, power, optical). The signal is received via sensors and decoded to retrieve the original, confidential data. The receiving device is assumed to be unsecured and, thus, is able to transmit the confidential data to the external world. The receiving and transmitting entities can be two separate IoT devices [32], two personal computers [31], two cores within a multi-core processor [37], or even two FPGAs in a data center [38].

#### 2.1.2. Methods of Encoding Data

There are three primary encoding schemes that are commonly used in covert channels: (1) on–off keying (OOK), (2) Manchester encoding, and (3) Binary Frequency Shift Keying (BFSK) [31,33,34,37].

**Figure 3:**
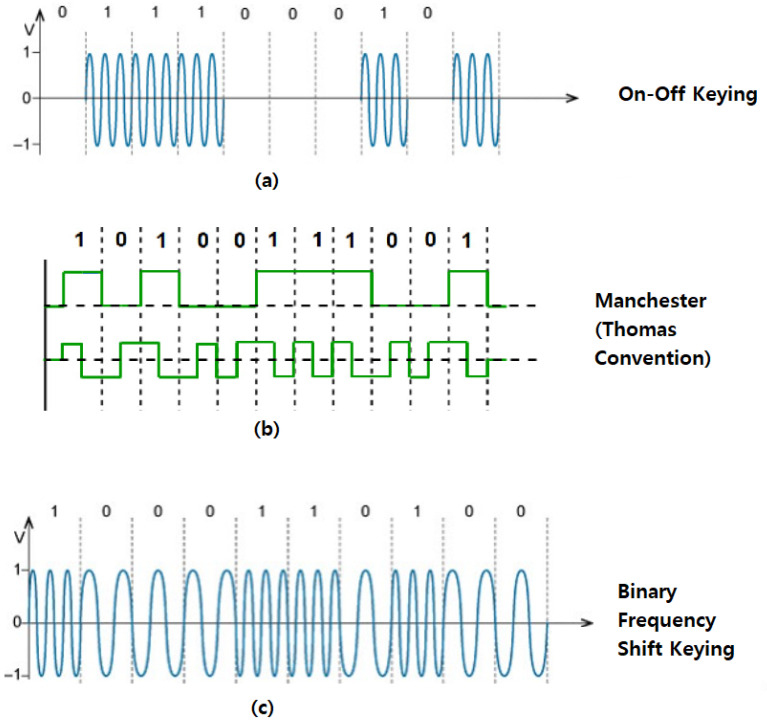
Common encoding methods for covert channels: (**a**) On–off keying, (**b**) Manchester Encoding, and (**c**) Binary Frequency Shift Keying.

On–off keying is the simplest form of general amplitude-shift keying (ASK) modulation [34]. The presence of a signal, or carrier wave, for a certain duration encodes a logical one (“1”), while no signal or carrier wave for the same duration encodes a logical zero (“0”), as shown in Figure 3a.

Manchester encoding is a scheme where each binary value is sent using two physical bits, with the transition occurring in the middle of the original bit. Each data bit is either low then high, or high then low, for equal time [34]. Depending on the convention used (Thomas or IEEE 802.3 [39]) a logic 1 can either be represented by a logic low followed by a logic high or vice versa, as depicted in Figure 3b. Manchester encoding’s transfer rate is half of that of OOK, since it uses two physical bits for each logical bit. This type of encoding is considered more reliable because of the redundancy of each transmitted bit [34].

In binary frequency shift keying (BFSK), frequency is modulated at varying rates to produce a logic “1” or logic “0” [34]. Binary Frequency Shift Keying uses a pair of discrete frequencies to transmit binary information. The instantaneous frequency of the carrier is switched between two values in relation to the binary values being transmitted, as illustrated in Figure 3c.

### 2.2. Covert Channel Evaluation

The channel capacity of covert communication typically refers to the maximum amount of information that the channel can transmit per unit time, usually measured in bits per second (bps). The Trusted Computer System Evaluation Criteria (TCSEC) [40] states that a channel bandwidth more than 100 bps is considered a high-bandwidth channel. Physical covert channels have been shown to have widely varying bandwidths, ranging from a few bps to kilobits per second (kbps). Table 1 summarizes the important characteristics of various types of physical covert channels. Generally, thermal covert channels are at the lower end of the spectrum, at approximately 10 bps. The bandwidth of power covert channels is slightly higher at a few hundred bps [32,38], and electromagnetic covert channels have the highest bandwidth on the order of multiple kbps [31]. Generally, the bandwidth decreases if the covert channel is established between two separate devices and not between two compute elements within the same device. Another metric commonly used to describe covert channels is bit error rate (BER), which is the number of incorrect bits transmitted, divided by the total number of transferred bits, over a period of time. Physical covert channels aim to have a BER that is as low as possible, typically below 2%, as listed in Table 1 [32,33,38,41]. Although both bandwidth and BER are able to measure and quantify the performance of the side-channel, no insight on the covertness is provided by these metrics. Carrara et al. proposed using metrics, such as steganographic capacity, to remedy this issue [5]. Steganographic capacity refers to the maximum amount of data that can be covertly transmitted before the likelihood of detection.

## 3. Types of Physical Covert Channels

Physical covert channels can be classified into five main categories: electromagnetic, power, acoustic, thermal, and optical, as previously shown in Figure 1. A summary of bandwidth, bit error rates, example works, and detectability of various types of physical covert channels is listed in Table 1, as discussed above.

Covert channels can be created from optical emissions of light-emitting diodes (LEDs) in many types of devices, such as monitors [45], keyboards [46], hard drives [34], etc. As long as line-of-sight is maintained, optical covert channels could have a very high transmission rate. However, it is very unlikely for a secure computer to be in an environment that would also have a malicious, undetected camera to act as a receiver and for the flashing LEDs to go unnoticed. Similarly, acoustic covert channels can be created from computer speakers [30,44], or even the noise from fans [47]. Acoustic channels generally have lower bandwidth and their waves do not travel very far. Additionally, an observant user may be able to notice the presence of audible noise, which can make this type of covert channel detectable. Since optical and acoustic channels are relatively easier to detect, these covert channels are not discussed in this work. Thermal (Section 3.1), power (Section 3.2), and electromagnetic (Section 3.3) covert channels are summarized with specific novel methodologies discussed in detail in the following sections.

### 3.1. Temperature-Based Channels

Modern electronic devices feature easily accessible temperature sensors that are typically used for dynamic thermal management [48,49]. These sensors were recently shown to be a potential security threat, since otherwise isolated applications can exploit them to establish a thermal covert channel (TCC) and leak restricted information. Temperature can be used as a covert channel within the same core of a processor (via leveraging multiple threads) [37], between different cores of a multicore processor [37], or even between adjacent desktop computers [33].

**Figure 4:**
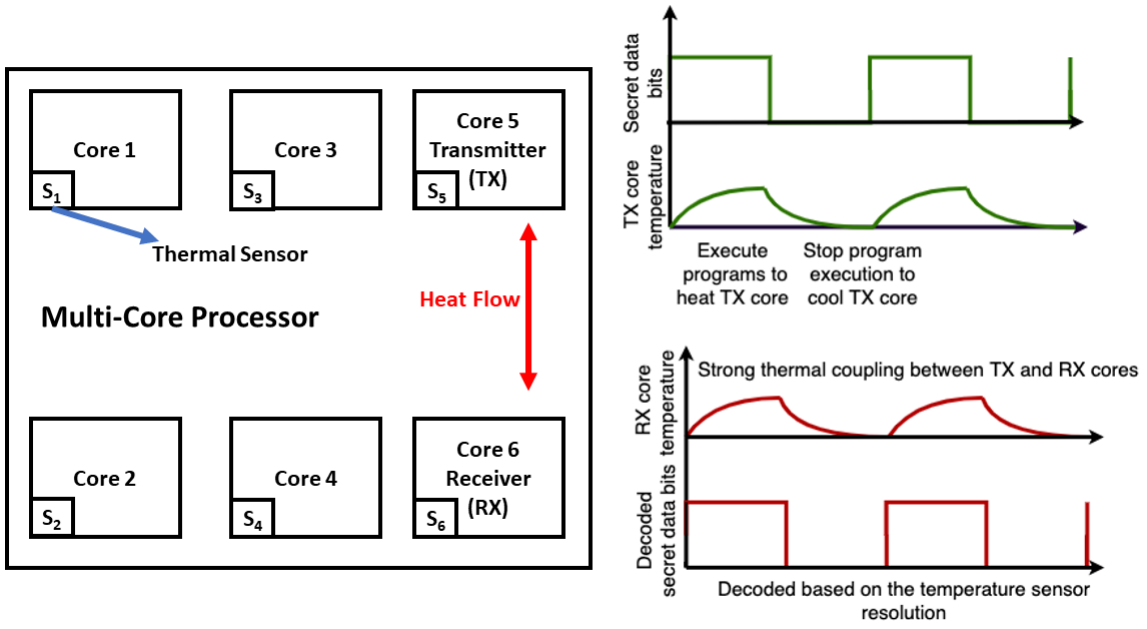
Temparture-based covert channels in spatially separated cores of a processor [51].

The threat model of a TCC in a multicore processor is as follows: the application at the transmitting core controls the power consumption (and, consequently, the temperature) of that core, resulting in the temperature of the transmitting core being encoded with the secret data, as shown in Figure 4. An application running on the receiving core has access to the temperature sensor and reads the encoded temperature profile and decodes the signal to retrieve the secret data [37]. This type of attack can be accomplished fully remotely since the attacker does not need direct physical access to probe or measure the IC [50].

The bandwidth of TCCs varies widely, depending upon the location of transmitter and receiver. For example, if the communication is between discrete desktop computers, the bandwidth is, typically, a few bits an hour [33]. The upper bound of the capacity of a temperature covert channel is, theoretically, estimated as 300 bps for the same core, and on the order of 50 bps for two adjacent cores [37]. Additionally, the TCCs are verified experimentally with Manchester encoding on a laptop, server, and cellphone, where the bandwidth reaches up to 90 bps at a cost of 10% BER [37]. TCCs are highly practical for attackers who prefer a fully remote attack, enabled by thermal sensor information. An important disadvantage to TCCs is their relatively low bandwidth, compared to power and electromagnetic covert channels.

#### BitWhisper

BitWhisper is a methodology that allows two nearby computers to communicate with each other, even if both computers are air-gapped [33]. It is possible to transmit data to another computer that is located in close proximity by measuring and analyzing the temperature changes generated by running a GPU stress tester, such as FurMark [52] and prime65 [53], and a CPU stress tester, prime95 [53], which calculates Mersenne primes.

The exchange of data between two computers is demonstrated within a distance of 1–40 cm from each other utilizing the Bitwhisper covert channel [33]. The communication channel between the computers can be bidirectional. BitWhisper has a relatively low bandwidth, of only 8 bits per hour, compared to other physical covert channels [33]. This work observed that a normal workload did not effect temperature of a desktop significantly, thus making it possible to use a computer as a receiver during normal operation. While BitWhisper does propose a novel attack that requires no additional hardware, it is impractical, due to both the very low bandwidth and the required close proximity (tens of centimeters) of the devices.

### 3.2. Power-Based Channels

**Figure 5:**
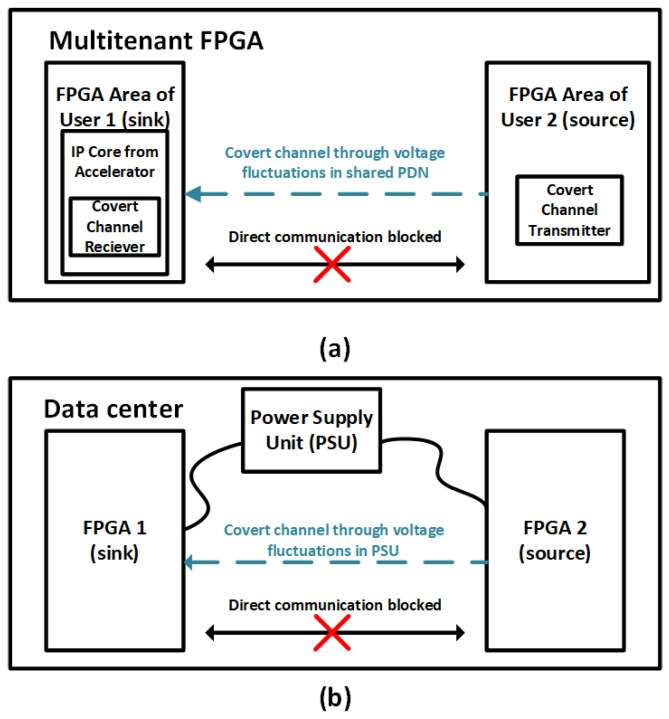
Voltage-based covert channels as a threat between (**a**) a multi-tenant FPGA [54], (**b**) two FPGAs in a data center sharing the same power supply unit (PSU) [38].

Power, and, subsequently, supply current consumption, can be used to establish a covert channel through an on-chip power delivery network or even an electrical outlet that a device is plugged into. Similar to thermal channels, malicious software on the transmitter runs CPU intensive instructions in order to encode the data into voltage drops along the power delivery network. Figure 5a shows an example of an attack model of a power covert channel occurring on a multi-tenant FPGA, where voltage fluctuations along the shared power delivery network are caused by the transmitter [54]. Custom logic in the receiver, such as ring oscillators (ROs), is able to detect the fluctuations and decode the data being transmitted. Similarly, Figure 5b shows a power covert channel occurring in a data center between two FPGAs that share the same power supply unit (PSU) [38]. Activity in FPGA 2 causes fluctuations in voltage supplied by the PSU, which can be detected by FPGA 1 (a malicious user in the data center).

#### 3.2.1. PowerHammer

PowerHammer is the first work to introduce the power-based (current flow-based) covert channel [32]. In this work, binary information is encoded by increasing and decreasing the current flow, which is then propagated through the power lines, and intercepted by an attacker. The receiver in PowerHammer is a current probe connected to a small computer (for demodulation). The probe is attached to the power line (line-level attack) feeding the computer at the electrical outlet or even the main electric panel (phase-level attack).

Adjusting CPU workload controls the power consumption, i.e., overloading the CPU with jobs results in more current consumption. PowerHammer regulates the workload of each core separately in order to increase stealthiness of the attack (cores being used for normal operation are not interrupted). PowerHammer also controls the amount of cores used in the attack, which gives flexibility to manipulate the amplitude and modulate the current consumption to encode data. A carrier wave is generated by applying a workload at full power consumption for half a period and no power consumption for the other half, where the time period determines the frequency of the generated carrier wave. Frequency-shift keying modulation is used for encoding data. Data is transmitted in frames consisting of 44 bits, with a preamble, payload and cyclic redundancy check (used for error detection) [32]. The transmitting program (that controls the workload of the cores) requires no special or elevated privileges (e.g., root or administrative) and contains basic CPU operations, which do not imply malicious behavior, therefore making it difficult to detect.

The authors measured the current consumption of a PC, a low power IoT device (Raspberry Pi), and a server. They determined that the PC was highly susceptible to this type of attack. The current probe used was a split core current transformer connected to a laptop computer. The probe was secured around the power line directly connected to the device or inside the main electrical service panel of the entire floor. The probe measured the amount of supply current passing through. With the malicious program changing the workload from 2 cores to 8 cores, the amount of current drawn increased from 2.5 mA to 19 mA in the power-line level attack [32]. The PC maintained transmitted bit rates of 333 bps, 500 bps and 1000 bps without errors (0% BER) [32]. The Raspberry Pi only achieved low bit rates of 1 bps and 10 bps with a BER of 1.9% and 4.8%, respectively [32]. The results showed that desktop computers could be used to transmit a considerable amount of information (such as images, documents) and that low power devices (like the Raspberry Pi) were relevant for the transmission of small amounts of data (such as passwords). Phase-level attacks were demonstrated to have higher amounts of interference and, thus, the bit transmission rate was much lower, averaging approximately 10 bps for a laptop [32].

#### 3.2.2. C${}^{3}$APSULe

Users renting FPGAs from cloud providers assume that their designs are securely separated from users using other FPGAs within the same data center. However, C${}^{3}$APSULe shows that this assumption does not hold, due to the leakage of shared power supply units (PSUs) [38]. A physical covert channel attack is introduced between FPGAs that are powered by the same PSU [38]. Furthermore, if the PSU also powers the host computer, CPU-to-FPGA and GPU-to-FPGA covert channels can also be created. This work used ring oscillators to sense and stress the source and sink FPGAs. Voltage and temperature monitors are inaccessible to end-users of cloud FPGAs, which means non-invasive detecting of voltage fluctuations is nontrivial. However, ring oscillators can be implemented to detect voltage changes, because the reconfigurability of FPGAs is still available to the attacker. The varying supply voltage changes the RO frequencies, allowing the attacker to correlate processor workload with RO frequency. Thus, no invasive measurement setups or probes were required for this methodology because of the designed ring oscillators. Similarly, reference [54] used Time-to-Digital Converter-based (TDC-based) voltage sensors instead of ring oscillators to detect the supply voltage fluctuations.

Note that the voltage regulator within the printed circuit board of the receiver should be overloaded in order to detect transmissions by the source (transmitter) FPGA. This requirement is achieved by introducing “stressor” ROs within the receiver. In C${}^{3}$APSULe, the ROs are implemented using lookup tables, consisting of 1 inverter and 3 buffer stages. From the sink side, there are ROs that make up the receivers and there are additional ROs that stress the voltage regulator of the sink FPGA. Once the stressors are turned on, the transmitters are enabled for measurement periods dependent on the data being encoded. This causes fluctuations in the PSU, which the receiver measures by counting the RO signal transitions in a fixed measurement interval. The RO counts are averaged over repeated measurements and Manchester encoding is used to minimize the impact of noise in the system. The methodology was implemented with 2 different FPGA boards. Cross-FPGA communication was shown to have ∼4% BER when there were 10 sets of transmitters, where each transmitter had 800 ROs [38]. Depending on the FPGA board, the amount of LUT resources used varied from 3.4% to 16.6% just for the source side implementation (500–2500 ROs total) [38]. The channel capacity of this methodology was shown to be 3 to 6 bps [38]. Similar bandwidths and BER are achieved when using a CPU and GPU to transmit data with stress tests. While this is a novel and remote covert channel attack, the bandwidth is relatively low. Furthermore, C${}^{3}$APSULe relies on the assumption that cloud FPGA providers do not recognize that attackers implement designs with up to 10,000 ROs with the intent to sense voltage drops.

### 3.3. Electromagnetic Radiation-Based Channels

Electromagnetic (EM) signals can be used as a medium for physical covert channels, with the unique ability to travel through many physical obstacles (i.e., concrete walls) with negligible energy loss. BitJabber is a high bandwidth covert channel that uses the spectra of EM waves to transmit data between air-gapped devices [31]. The sender creates the covert channel through memory accesses to modulate the electromagnetic signal generated by the clock signal of the DRAM chip. It was determined earlier in [55] that accessing memory results in unwanted side-channel information leakage, specifically with the same frequency as the memory accesses. For example, memory accesses with an execution time of approximately 350 ns result in an EM spectra with raised energy at multiples of that frequency (2.86 MHz). After measuring the EM signal on the receiver side, the data is extracted by observing the spectra at these known frequencies, which correspond to either a bit 0 or 1 being transmitted. The major contribution of Bitjabber is its potential for such a high bandwidth, while still being able to penetrate thick concrete walls to an adjacent room. It was shown that BitJabber was implemented with two desktop computers. A log-periodic type of antenna was used to collect the EM signals around the DRAM clock frequency (400 MHz to 1 GHz) [31]. The data was collected in a typical office environment, which had multiple sources of background noise (such as radio stations, cell towers, other components within the desktop, and wires inside the walls). Experiments were performed with two scenarios: (1) the antenna was placed adjacent to the computer to receive the strongest EM waves from the DRAM clock signal and (2) the antenna was placed in a neighboring office that shared a 15 cm thick concrete wall. For the experimental setup with the antenna adjacent to the computer, with OOK modulation at a bandwidth of 100,000 bps, there was only a 0.4% bit error rate [31]. Utilizing BFSK modulation decreased the bit error rate to 0.25% at the same bandwidth of 100,000 bps [31]. Bitjabber is a practical covert channel because of its high bandwidth. Additionally, it can be relatively difficult to detect because the memory accesses required to encode the data can look like normal operation. The capability of EM waves to pass through walls means that it is less obvious to observant users, as compared to optical or acoustic covert channel attacks.

## 4. Countermeasures against Covert Channel Attacks

There are multiple developments related to countermeasures of covert channels. Major countermeasures include the following: shielding through physical means, to block the transmission of data (Section 4.1); jamming, which involves the injection of noise into the system to make the channel transmit incorrect data, and, thereby, increasing BER (Section 4.2); and runtime detection, which involves monitoring of the system for anomalous/unusual activity (Section 4.3).

### 4.1. Shielding

One type of countermeasure for physical covert channels is adding shielding to attempt to block the transmission medium chosen. For example, Faraday cages are a common proposed countermeasure for electromagnetic covert channels in order to attenuate the signal. TEMPEST is a shielding standard developed by NATO and the National Security Agency (NSA) that requires systems to be protected with “a 100 dB insertion loss from the frequencies of 1 KHz to 10 GHz” [56]. However, note that there are techniques that are able to establish covert channels, despite various types of shielding, by manipulating the shape of the frequency spectrum [57] or focusing on the lower end of the frequency spectrum [36]. While shielding does provide a physical impediment to the communication medium, the ever-evolving nature of attacks has shown that relying on passive methods does not maintain a guarantee of security.

### 4.2. Jamming

Another type of countermeasure is jamming, which involves introducing noise to a system in order to sufficiently increase the BER of the channel, thus making the transmitted data useless. Thermal noise is introduced in TCCs where the frequency band of the noise overlaps with the covert channel data transmission frequency [58]. However, this broad spectrum jamming fails to interfere with a channel that is enhanced by exploiting frequency-hopping spread spectrum (FHSS) [59]. FHSS is a technique where the frequency of the transmitted signal changes over time in order to avoid interference. Both the transmitter’s and receiver’s frequency hopping pattern are synchronized. An enhanced jamming model that periodically scans the frequency spectrum for an attack and injects noise corresponding to that frequency band, in order to thwart TCCs enhanced by FHSS, was introduced in [58]. Compared to other countermeasures jamming is highly inefficient, because it requires high intensity instructions to be executed for the CPU to generate these thermal waves, thus wasting significant power [58]. Furthermore, jamming implies that the core is not able to perform normal tasks during this time. Similarly, software can either execute power intensive instructions on the electronic device to introduce noise to power-based covert channels [32], or perform irregular memory accesses to increase error rates for electromagnetic covert channels [31].

### 4.3. Runtime Detection of Covert Channels

One of the most important countermeasures for covert channels is dynamically monitoring the host system in order to detect the presence of unauthorized data transmission. Typically, detection methods can be classified as threshold-based monitoring and machine learning-based methods. Threshold-based monitoring refers to the medium/signal of choice (instructions per cycle, power consumption, and data from thermal sensors) being observed during normal operation and a baseline being set. The system is monitored to see if this threshold is surpassed, which would indicate malicious behavior (the presence of the covert channel). Machine learning methods utilize the data from monitoring the system to train a neural network that can perform classification and, thus, determine the presence of a covert channel. These techniques are summarized in the following subsections.

#### 4.3.1. Threshold-Based Monitoring

Threshold-based methods involve monitoring system activity and determining a threshold that defines normal operation. If the amplitude of the signal in question is higher than this threshold value, a covert channel is suspected. The major challenge is determining a threshold value that accurately detects covert channels without triggering false positives (i.e., high detection rate and low false positive rate). Specifically, a monitoring system of RO-based voltage sensors and frequency counters in the power delivery network can be used to determine voltage drops in multi-tenant FPGAs [60]. The frequency of the RO-based sensor decreases in response to a voltage drop. A calibration procedure is used to correlate the change in frequency of the RO to voltage drop. Although this technique is evaluated to prevent attacks that cause supply voltage instability (thereby crashing the FPGA), it can be modified by changing the threshold voltage to detect power covert channels in FPGAs. However, the challenge remains in choosing the correct threshold value that minimizes the amount of false positives from normal operation. The selection of a feasible threshold value is challenging, due to the wide range of applications that can be potentially executed on a device.

One threshold-based technique for detecting TCCs involves analyzing the power spectrum of the temperature signal in the frequency domain [61]. This technique involves using a band-pass filter at various frequency steps, which can be time-consuming. Another method involves analyzing the frequency spectrum of the CPU workload of each logical core to detect TCCs, which eliminates the need for a band-pass filter [62]. This method involves quantifying the CPU workload using instructions per cycle (IPC) and obtaining the power spectrum of the IPCs. If the maximum amplitude of the spectrum exceeds a predetermined threshold, it is assumed that a covert channel is present. To optimize data transmission through a covert channel attack, it is suggested to avoid the frequency range of 0 to 10 Hz, as this range corresponds to the power spectrum of typical applications that the core is expected to execute [63]. As a result, the detection method in [62] focused on the frequency range from 10 Hz to 500 Hz.

**Figure 6:**
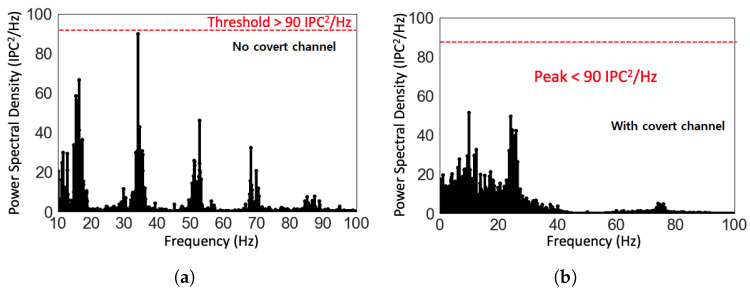
Power spectral density (PSD) of instructions per cycle (IPC) simulated during the execution of (**a**) random applications (sequentially) from SPLASH-2 and PARSEC benchmark suites on an Intel Haswell processor core (no covert channel) (**b**) a low power benchmark, raytrace, encoded with the secret data (i.e., with TCC).

Previous work has demonstrated that typical low power programs (such as raytrace [64]) can be used to establish high bandwidth TCCs in scenarios where there is significant thermal coupling between the cores [65]. Existing detection techniques fail to accurately detect these kinds of TCCs because less resources (e.g., IPC) are used than the calibrated threshold. The power spectral density of IPC during normal usage was simulated by executing random applications (sequentially) from SPLASH-2 and PARSEC benchmark suites on an Intel Haswell processor core. As shown in Figure 6a the maximum amplitude of the power spectrum was 90 IPC${}^{2}$/Hz, which would then be defined as the threshold. Figure 6b shows that the power spectral density of IPC with a covert channel was less than the defined threshold of 90 IPC${}^{2}$/Hz. Therefore, the TCC would not be detected. Machine learning techniques have been proposed as a solution to mitigate the drawbacks of threshold-based detection.

#### 4.3.2. Leveraging Machine Learning Techniques

Machine learning has been used as a method to detect anomalies and classify whether processor activity is suspicious (i.e., the presence of a covert channel communication), particularly for cases where signal amplitudes are smaller and threshold-based techniques would not be sufficient, as described above [62]. It was found that TCC signals have multiple side lobes of high amplitudes that can be used for detection [66]. An artificial neural network classifier was developed and trained for TCC detection. The training data consisted of thermal signals over a period of 2 s, sampled at 1000 Hz, that were then transformed into the frequency domain (10 Hz to 500 Hz) with a discrete Fourier transform [66]. After training, this classifier was used during runtime to infer TCCs. The global manager ran a detection cycle to check for a TCC, where the spectrum of the IPC signals of each logical core was extracted. The IPC spectrum was used during detection, instead of actual thermal signals, since they were correlated, i.e., an increase in IPC resulted in an increase in temperature. The proposed detection method using artificial neural networks was able to achieve a detection accuracy of 99%, even for TCCs with the lowest amplitudes (stealthiest) [66]. Additionally, it was shown to cost less in runtime overhead (<0.187%) and energy overhead (<0.072%), as compared to jamming-based countermeasures [66]. While this work demonstrated high detection accuracy, the effect of the location of the receiving and transmitting cores on the accuracy was unexplored. Additionally, the effect of varying amounts of noise from other cores performing normal or CPU intensive operations was not quantified. Finally, an analysis of how accuracy changes with varying system size (total number of cores) would provide a metric on how generalized the proposed neural network classifier is.

Similarly, a three-layer convolutional neural network (CNN) was developed to detect electromagnetic covert channels in [57]. The CNN was trained by using labeled EM spectra and legitimate/expected system processes. To test the capability of the neural network, white noise was added to the testing spectrum to simulate a jamming-based countermeasure (to increase attenuation). It was shown that the CNN could identify covert channel signals with 99% accuracy when there was less than 12 dB of attenuation. After this threshold, there was a drastic decrease in accuracy. For example, detection accuracy dropped to 60% at 16 dB attenuation.

## 5. Covert Channel Attacks in 2.5D/3D ICs

Even though hardware security in 3D ICs has received some attention [67,68,69,70], covert channel attacks in 2.5D-/3D-based integration are largely unexplored. The most related existing works are on thermal side-channels [51,71,72]. Other hardware security studies related to 2.5D/3D integration primarily focus on supply chain vulnerabilities, such as the presence of malicious chiplets (in the form of both software and hardware Trojans) and IP piracy [73,74,75,76]. For example, an active interposer in a 2.5D system can be leveraged as a root-of-trust to host a hardware security module and various security features, assuming that the interposer is designed and fabricated by trusted entities [77]. This approach, however, is highly vulnerable to semi-invasive and invasive physical attacks, since the interposer is relatively accessible to a malicious user who can, potentially, bypass these security features while maintaining the functionality of the chiplets. Furthermore, these hardware security features do not protect the 2.5D/3D IC against malicious end users who can exploit tightly coupled chiplets to establish efficient covert channel communication. Since individual compute units (i.e., chiplets) are expected to be much smaller in 2.5D/3D integration, the impact of thermal noise from other chiplets is be weaker, exacerbating the security threat caused by such covert channel attacks. A concise introduction to 2.5D/3D integration is provided in Section 5.1. Existing covert channel attacks in 2.5D/3D ICs are discussed in Section 5.2. A power covert channel attack model is proposed in Section 5.3. Finally, design-time techniques, as potential countermeasures, are discussed in Section 5.4.

### 5.1. 2.5D/3D Integration

The number of commercial applications that utilize advanced packaging technologies has been increasing. These technologies include interposer, or interconnect, bridge-based 2.5D integration [78], high density organic substrates [79], TSV-based 3D integration with active interposer [80], fan-out wafer-level packaging [81], and hybrid bonding [82,83]. Despite important differences in physical characteristics and cost, each of these emerging packaging technologies enables dense integration of chiplets within a single package [84]. Chiplet-based integration has the potential to provide heterogeneous systems, where chiplets, with diverse functions, can be fabricated with different technology nodes [85]. Having many, smaller chiplets (instead of a large monolithic die) increases yield and, potentially, decreases the overall cost [86,87]. Furthermore, in 2.5D/3D integration, since chiplets are tightly coupled and interconnect density is high, conventional parallel interconnects can be used, which consume less power at lower interconnect latency as compared to serial interconnects [88]. These advantages are particularly important for emerging data-centric applications in domain specific computing, such as machine learning and Internet-of-things. Despite these promising advantages, dense 2.5D/3D integration of heterogeneous chiplets brings new and largely unexplored security challenges, such as physical covert channels.

### 5.2. Existing Works

Although covert channel attacks in 2.5D and 3D systems are largely unexplored, there are several recent works on power covert channels in 2.5D FPGAs and TCCs in 3D ICs, as described below.

#### 5.2.1. Power Covert Channels in 2.5D FPGAs

FPGAs are an example application domain of 2.5D integration technology, with commercial FPGAs consisting of multiple dies on the same package [89,90]. Giechaskiel et al. demonstrated that sensing the changes in supply voltage between separated dies within an FPGA chip was possible [91]. The same receiving and transmitting RO setup was used as [38]. However, the attack took place within the same FPGA. Similarly, this attack was also fully remote, because attackers did not have physical access to cloud FPGAs. The authors demonstrated that, as transmitter size (amount of ROs) increased from 100 to 500, the BER decreased from 25% to 0.1% [91]. Since this covert channel took place on-chip, a much higher bandwidth of 4.6 Mbps was achieved with Manchester encoding at a BER of 2.4% [91]. However, the overhead to reach this BER and bandwidth was relatively high. Specifically, there were 12 transmitters, each consisting of 2000 ROs [91]. The overhead for sensing the supply voltages on the receiver side was much lower. Specifically, there were 5 receivers, each consisting of 5 ROs [91]. Unfortunately, the area and LUT usage was not quantified, but the sheer amount of ROs required to make this attack successful could make this attack more detectable.

#### 5.2.2. Thermal Covert Channels in 3D ICs

**Figure 7:**
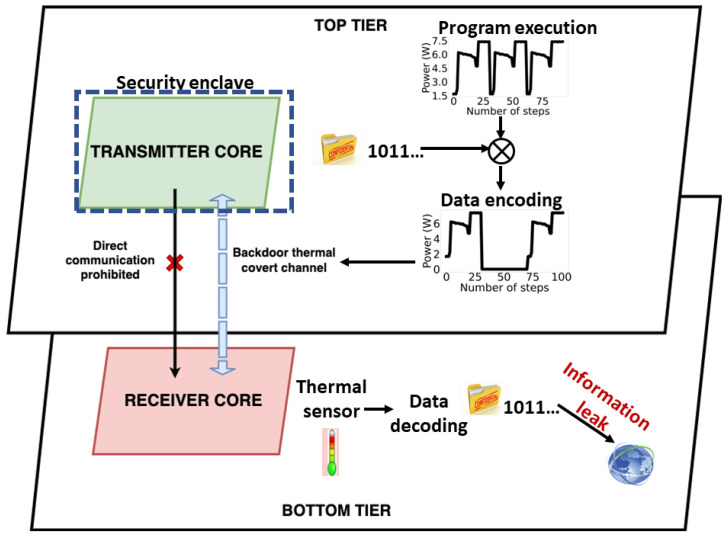
Attack model of a thermal covert channel between a secure and insecure chiplet in a 3D IC.

It was recently demonstrated that highly reliable TCCs could be created through the use of low-power programs on 3D ICs [65]. These channels are established when the source and sink nodes, which are located in different tiers of a 3D IC, are placed in close proximity to each other. The close proximity of cores in a 3D multicore processor enables strong vertical thermal coupling, which can increase the rate of covert communication by a factor of 3.4, compared to covert communication in traditional 2D integrated circuits [65]. This strong vertical thermal coupling facilitates the use of typical low power benchmark applications to establish high bandwidth covert communication in 3D ICs [92]. The TCC attack model in 3D ICs is shown in Figure 7. The attacker executes an app within the secure chiplet with access to confidential data. Due to the security policy of this chiplet, this data cannot be accessed by the external world. However, the attacker establishes a TCC by controlling the execution of a program within the secure chiplet. Specifically, the transmitting app raises and lowers the power consumption (and indirectly the temperature) of the transmitting chiplet via a program. Thus, the temperature profile of the transmitting chiplet is encoded with secret data which couples to the receiving chiplet, due to dense 2.5D/3D integration and elevated temperature levels. Since the receiving chiplet is not within a security enclave, a low-activity app running in this chiplet has access to a temperature sensor and can read the encoded temperature profile [37]. The app then decodes the temperature profile to retrieve confidential data. The attacker does not need physical access to the system, since the entire attack can be completed remotely [37,50].

This work presented results on TCCs in both monolithic inter-tier, via MIV-based monolithic 3D (Mono3D), and through-silicon, via 3D-based (TSV3D) technologies [65]. The authors demonstrated that it was possible to transfer data at 200 bps with a BER of less than 1% in both Mono3D and TSV3D [65]. It was concluded that the bandwidth of TCCs in 3D ICs was relatively unaffected by thermal interference when other cores were active. Alternatively, in traditional 2D processors, the bandwidth degraded by 12% and the BER also increased from less than 1% to 3% when there was thermal interference [65]. Consequently, the TCC was relatively more robust in 3D ICs. To reduce the thermal coupling between cores in 3D integrated processors, the authors demonstrated that it was possible to decrease the vertical overlap between secure and insecure cores. The TCC bandwidth between non-overlapping cores in Mono3D and TSV3D processors was reduced by up to 62% and 58%, respectively, compared to TCC bandwidth between fully overlapping cores [65]. A 50% overlap between transmitting and receiving cores was also explored. The results showed an approximately 16% degradation in bandwidth (∼160 bps) [65]. The authors also showed results on moving the transmitter core closer to the heat sink on the bottom tier and placing the receiver on the upper tier above the transmitter. Since the dominant heat flow was toward the heat sink, the transmitter and receiver temperatures were almost identical, thereby increasing the bandwidth of the covert channel by approximately 10% [65].

Finally, the authors quantified the impact of having an additional tier between the transmitting and receiving cores. This scenario was investigated by partitioning an Intel Haswell processor into 4 tiers. It was determined that the bandwidth remained the same (when there were no other active cores) in Mono3D technology, because the cross-sectional layers were sufficiently thin and the vertical thermal coupling remained strong (despite an additional tier between the transmitter and receiver). In TSV3D technology, the bandwidth increased by 10%, primarily due to greater temperatures, since the resistance to heat sink increased in a 4 tier stack.

In this 4 tier system, a noise application executed in the tier directly below the receiving core resulted in a lowered bandwidth of 100 bps with a BER of approximately 2.5% [65]. These results indicated that the vertical thermal coupling in 3D systems was strong enough that additional tiers did not prevent covert channel communication. However, this strong coupling could also facilitate the development of effective jamming-based countermeasures.

### 5.3. Potential Covert Channel Attack Model in 2.5D ICs

General covert channel attack models, described in Section 2.1.1, also apply to 2.5D ICs, due to dense integration of chiplets within the same package. Here, we propose a slightly different attack model that has the potential to yield high bandwidth with low BER. The proposed attack model exploits the interposer layer of 2.5D ICs, since this is relatively accessible to users. Specifically, power signals were leveraged to establish covert communication and, therefore, bypass existing hardware security measures proposed in the literature for 2.5D systems. We assumed that at least one of the chiplets, referred to as the transmitter chiplet, operated in a secure zone/enclave [1,93,94,95] and had access to confidential information protected by existing security features. The receiver chiplet operated within the insecure zone and had external connectivity. The transmitter and receiver were not permitted to communicate, due to the security constraints.

**Figure 8:**
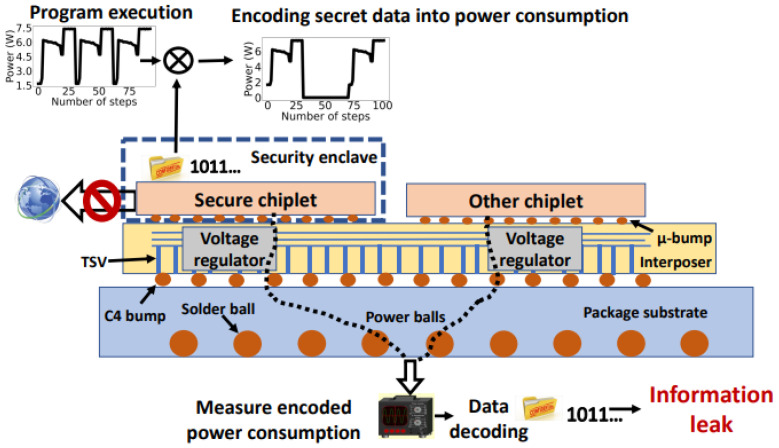
Attack model of a power covert channel between a secure chiplet and user/attacker.

As shown in Figure 8, in this attack model, the user is assumed to have physical access to the 2.5D chip and equipment to measure power consumption. The attacker executes an app within the secure chiplet to raise and lower the power consumption, thereby encoding the confidential data into the power profile of the transmitting chiplet. Then, rather than monitoring the temperature of another chiplet, the attacker measures the total power consumption of the system. Since the total power is correlated with the power consumption of the secure chiplet, the attacker can decode this signal to retrieve confidential data. Note that the power due to other chiplets behaves as “noise”. The attacker can perform frequency domain analysis to filter this noise. Alternatively, the attacker can isolate the voltage regulator of the secure chiplet. Note that in chiplet-based integration, it is highly common for each chiplet to have dedicated regulators [96]. These regulators are typically placed within the interposer to save area and realize passive devices with high quality factors [97], as shown in Figure 8. This technique, however, introduces an important security vulnerability by providing a more direct approach for power covert channels. Specifically, an attacker can perform a semi-invasive attack that probes the active interposer and isolates the voltage regulator of the secure chiplet. Thus, the attacker can accurately measure the power consumed only by the secure chiplet, potentially producing a higher bandwidth channel with low BER.

These covert channel attacks pose serious security threats because they are potentially more powerful than traditional power side-channel attacks where the total measured power is correlated with a power model that relies on a single intermediate signal [98]. In side-channel attacks, this weak correlation can be mitigated by a large set of existing works that reduce the dependence of power on input signals [99,100]. A disadvantage of the proposed power covert channel attack in 2.5D ICs is that it is not remote and requires a physical probe. It should be noted that intrusion detection methods have been developed to be able to sense a malicious measurement probe in the context of side-channel attacks [101,102,103,104]. Such techniques are applicable to the proposed attack model as well.

### 5.4. Discussion on Mitigation Strategies

In chiplet-based integration, design time techniques to mitigate side-channels and covert channels are limited since chiplets are typically obtained as a standalone IP, where only certain information is available, such as I/O characteristics, area, power, and performance. Thus, the applicable design-time techniques are related to the floorplan/placement of the chiplets. Specifically, to mitigate TCCs, the floorplan should reduce potential temperature gradients between the chiplets, particularly between secure and insecure chiplets. Reduced temperature gradients decrease the horizontal heat flow, thereby mitigating the efficacy of covert channel communication. Similarly, the placement and in-package design of the secure chiplet should favor vertical heat flow toward the heat sink. In-package structures for heat isolation can also be considered. For power covert channels, an important design-time countermeasure is obfuscating the power delivery network, so that the attacker cannot isolate the regulator of the chiplet, via semi-invasive approaches that target the interposer (where regulators are typically placed). This obfuscation would be helpful, but not sufficient, since the total power would still be correlated with the secure chiplet power profile. Runtime covert channel detection techniques described in Section 4.3 would be required.

## 6. Conclusions

Physical covert channels are capable of subverting the established security policy of a device by transmitting data from a secure compute element to an insecure compute element. Physical covert channels accomplish this unauthorized data transmission through side-channel signals (such as temperature, power consumption, and electromagnetic waves). As such, they do not require physically shared resources between the compute elements, unlike host-based covert channels, such as caches, data path units, and memory controllers. In this paper, we first provided a background on methods to establish a covert channel, and then presented an extensive survey on, and perspective of, state-of-the-art physical covert channels in 2D ICs, and relevant countermeasures, including run time detection techniques. Additionally, the potential of covert channels in 2.5D/3D ICs, due to the increased coupling between chiplets, was discussed. We summarized existing recent works on covert channel attacks in 2.5D/3D ICs. Finally, we proposed power covert channel attack models for 2.5D ICs and discussed design-time techniques to mitigate covert channels in these emerging advanced packaging technologies.

## Figures and Tables

**Table 1:** Summary of characteristics of physical covert channels.

Type	Bandwidth (bps)	BER (%)	Detectability	Example Works
Thermal	0.002–300	1–11	Medium	[33,37]
Power	3–1000	0–5	Low	[32,38]
Electromagnetic	480–300,000	0.25–10	Medium	[31,41,42]
Optical	15–4000	1–8	High	[34,43]
Acoustic	0.25–230	1–2	High	[30,44]

## Data Availability

Data sharing not applicable.
